# Development and Validation of a Diabetic Retinopathy Referral Algorithm Based on Single-Field Fundus Photography

**DOI:** 10.1371/journal.pone.0163108

**Published:** 2016-09-23

**Authors:** Sangeetha Srinivasan, Sharan Shetty, Viswanathan Natarajan, Tarun Sharma, Rajiv Raman

**Affiliations:** 1 Shri Bhagwan Mahavir Vitreoretinal Services, Sankara Nethralaya, Chennai, Tamil Nadu, India; 2 Department of Preventive Ophthalmology, Sankara Nethralaya, Chennai-600 006, Tamil Nadu, India; Bascom Palmer Eye Institute, UNITED STATES

## Abstract

**Purpose:**

To develop a simplified algorithm to identify and refer diabetic retinopathy (DR) from single-field retinal images specifically for sight-threatening diabetic retinopathy for appropriate care (ii) to determine the agreement and diagnostic accuracy of the algorithm as a pilot study among optometrists versus “gold standard” (retinal specialist grading).

**Methods:**

The severity of DR was scored based on colour photo using a colour coded algorithm, which included the lesions of DR and number of quadrants involved. A total of 99 participants underwent training followed by evaluation. Data of the 99 participants were analyzed. Fifty posterior pole 45 degree retinal images with all stages of DR were presented. Kappa scores (κ), areas under the receiver operating characteristic curves (AUCs), sensitivity and specificity were determined, with further comparison between working optometrists and optometry students.

**Results:**

Mean age of the participants was 22 years (range: 19–43 years), 87% being women. Participants correctly identified 91.5% images that required immediate referral (κ) = 0.696), 62.5% of images as requiring review after 6 months (κ = 0.462), and 51.2% of those requiring review after 1 year (κ = 0.532). The sensitivity and specificity of the optometrists were 91% and 78% for immediate referral, 62% and 84% for review after 6 months, and 51% and 95% for review after 1 year, respectively. The AUC was the highest (0.855) for immediate referral, second highest (0.824) for review after 1 year, and 0.727 for review after 6 months criteria. Optometry students performed better than the working optometrists for all grades of referral.

**Conclusions:**

The diabetic retinopathy algorithm assessed in this work is a simple and a fairly accurate method for appropriate referral based on single-field 45 degree posterior pole retinal images.

## Introduction

Single-field fundus photography with interpretation by trained readers serves as a screening tool to identify patients with diabetic retinopathy (DR) for referral for further ophthalmic evaluation and management [1,2]. The ease of use, convenience, and ability to detect retinopathy favors single-field funds photography; however, the reported sensitivity values are less than ideal when compared with 7-standard field photography [3]. Nevertheless, it continues to be a screening method, especially when more numbers need to be screened [4]. In India, although the overall prevalence of DR is lower than western population, the absolute numbers to be screened is very high [5,6].

The major challenge in this model of screening is the training of graders. The conventional grading of DR by Early Treatment Diabetic Retinopathy Study (ETDRS)[7] and the International Clinical Disease Severity Scale for DR[8] needs understanding of the ETDRS scales and is more pertinent when multiple fields of retinal images are obtained. The aim of this study was to develop a simplified algorithm for referral of DR, which can be used by graders (physician/ophthalmologist/optometrist/trained paramedical worker) to grade single-field retinal images and to refer sight-threatening DR (STDR) for appropriate care. The accuracy of the algorithm was determined by a pilot study among optometrists, with the gold standard being the grading by a retinal specialist.

## Materials and Methods

The study was exempt from approval of Institutional Review Board of Vision Research Foundation, Chennai, India.(Letter attached)

### Development of the Algorithm

The International Clinical Diabetic Retinopathy and Diabetic Macular Edema Severity Scale[8] incorporated evidence on disease progression from the ETDRS and classified DR into 5 levels of severity based on retinal changes. The main advantage of the international severity scale was that the levels of severity were relevant to the clinical management plan for the patient. This facilitated communication between ophthalmologists and primary health-care providers. The problem with this scoring method is the learning curve involved in remembering the grades of retinopathy such as those which need immediate referral, 4–6 months follow-up, and yearly follow-up. To simplify, a red–green–yellow triage algorithm was developed based on the referral guidelines prescribed by the American Academy of Ophthalmology Retina/Vitreous Panel. Preferred Practice Pattern Guidelines [9].

**Fig 1:**
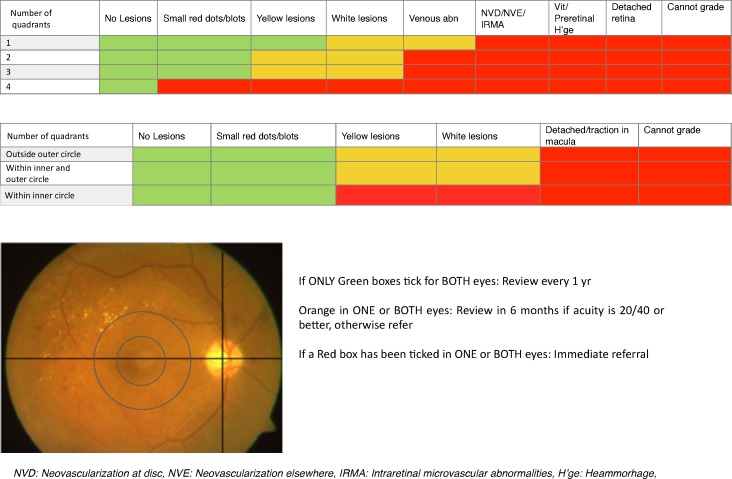
The red–green–yellow triage algorithm for quadrant-wise and macular evaluation, by using single-field 45 degree posterior pole photograph (lower left retinal photograph).

The grading of disease severity is based on the International Clinical Diabetic Retinopathy and Diabetic Macular Edema Severity Scale [8], while the referral guidelines are based on the American Academy of Ophthalmology guidelines [9]. The algorithm included the lesions of DR and number of quadrants involved for DR referral and location of lesions for diabetic macular edema referral (Fig 1). The single-field (posterior pole 45 degree) retinal image is divided into 4 quadrants by 2 imaginary lines. The horizontal line passes through the fovea and center of disc and the line tangent to the horizontal line passes through the center of the disc (Fig 1). For macular edema, 2 concentric imaginary circles are drawn at 1 disc diameter (1DD) and 2 disc diameter (2DD) from the center of macula.

The algorithm consisted of various DR lesions (listed below) and the number of quadrants (1–4).

The following are the various types of DR lesions to be identified.

#### Small red dots/blots

These include microaneurysms, dot-blot hemorrhages, and flame-shaped hemorrhages.

#### Yellow lesions

These include the deeper yellow lesions with sharp margins, which are the hard exudates.

#### White lesions

These include the more superficial, grayish white lesions with fluffy margins, and the cotton wool spots.

#### Venous abnormality or beading

These include venous dilation, loops, and beading.

#### IRMA/NVD/NVE

Intraretinal microvascular abnormality (IRMA) represent abnormal blood vessels, spider-web–like appearance (do not cross an artery or a vein and, therefore, appears only on one side of the vessel). Neovascularization at the disc (NVD) and neovascularization elsewhere (NVE) are new vessels from the surface of the retina or optic disc. Fibrous tissue accompanies the development of the NVD or NVE and sometimes may not have clearly evident new vessels.

#### Vitreous or preretinal hemorrhage

Preretinal hemorrhage is a boat-shaped lesion anterior to the retina with the blood settling in the space between the retina and vitreoretinal membrane. The hemorrhage has a flat top owing to the blood settling under the force of gravity. The hemorrhage in the vitreous cavity is the “vitreous hemorrhage.”

#### Detached retina

These include the tractional retinal detachment and combined tractional and rhegmatogenous retinal detachments.

As a preliminary step, the task involves noting down the presence or the absence of DR lesions and the number of quadrants having those lesions.

Similarly, the macular grading is done by noting the presence or absence of these lesions in (i) outside the outer circle, drawn at 2DD (ii) between the inner and the outer circles, drawn at 1DD and 2DD, respectively, and (iii) within the inner circle. Finally, an assessment of marked lesions is made to know which color boxes were marked.

If only green boxes are marked, for both eyes, a yearly follow-up is advised. This included no evidence of clinical signs of DR, mild non-proliferative DR (NPDR- with microaneurysms only) and no evidence of diabetic macular edema (DME). If any yellow boxes are marked, a review after 6 months is advised. This included lesions/signs of moderate NPDR- more than just microaneurysms but less than severe NPDR. However, if the visual acuity is worse than 20/40, an immediate referral is advised to look for other causes of reduced vision. If any red box is marked, an immediate referral is advised. This included findings of severe NPDR- more than 20 intraretinal hemorrhages in each of 4 quadrants, definite venous beading in 2+ quadrants, prominent IRMA in 1+ quadrant, PDR- neovascularization, vitreous/preretinal hemorrhage and DME.

### Pilot Study for Validation

The details regarding the pilot study was mailed to all eligible optometrists (n = 150) at a tertiary eye care institution. Eligible optometrists included working optometrists and optometry students in their final years of their optometry training. Participants were given a choice whether they would like to participate in the pilot study. Their participation and attendance was voluntary and they provided verbal consent. Ninety-nine participants confirmed their participation in the study for the study date. The study involved 50 posterior pole 45 degree retinal images with a mix of normal and all stages of DR from image database of a population-based study on DR. In the initial population-based study, informed consent was obtained from the participants regarding the use of the images for further studies. The institutional review board approved the study. These images were graded by single retina specialist for yearly follow-up, 6 monthly follow-up, and immediate referral. We included one retina specialist in the study so as to avoid inter-observer bias. The retina specialist is experienced with diabetic retinopathy grading and diabetic retinopathy screening for more than two decades.

A baseline information was collected from all participants related to demographics, highest qualification, number of years of optometric education, years of work experience as an optometrist, and information on whether they have seen a retinal image before and prior experience of DR grading. A summary of the baseline information is presented in Table 1.

**Table 1:** Background Details of the Participants.

Optometry participants (n = 99)	Mean (Minimum–Maximum)or n (%)
Age (years)	22 (19–43)
Men/women	13 (13)/86 (87)
No. of optometry students	49 (49)
No. of working optometrists	50 (51)
Highest qualification among optometrists (n = 50)	
Diploma in Optometry	1
Bachelors in Optometry	45
Master's in Optometry	3
Doctor of Optometry	0
PhD	0
No response	1
Average number of years of optometric education	4 (2–7)
No. of years of work experience as an optometrist (37/50 optometrists responded)	
<2	21
2–5	5
6–10	6
>10	5
Have you seen a fundus photograph? (n = 99)	
Yes	91
No	2
No response	6
Have you done diabetic retinopathy grading? (n = 99)	
Yes	10
No	79
No response	10

There were 50 practicing optometrists and 49 optometry students. A didactic lecture was taken for all the participants explaining the features of all the lesions of DR. The retinal images were projected on 2 wall-mounted white screens measuring 102 inches × 144 inches and on 2 Liquid Crystal Display (LCD) monitors measuring 22 inches × 41 inches (SANYO LCD-47XR7H; SANYO Electric, Co., Ltd., Japan), by a projector (SANYO PLC-XT35; SANYO Electric, Co., Ltd.) in an auditorium. The participants were seated such that the screens are clearly visible to them. A guided grading of 10 images was done to make them familiar with the grading process. Thereafter, 50 images were shown, and the participants graded the referral based on the algorithm on plain sheets. All participants graded 50 images; thus, there were 4950 expected referral responses (50 × 99). Ninety-nine participants actually gave 4780 responses (170 referral responses were not given). A comparison of these responses was done with the referral response by the retinal specialist.

### Statistical Analysis

Data were analyzed using the Statistical Packages for the Social Sciences (SPSS, version 14.0). Baseline information was analyzed as mean, standard deviation, and range or as proportions. The diagnostic performance of the optometry group was assessed by means of kappa statistics, sensitivity, specificity, area under receiver-operating characteristic curves (AUCs), and 95% confidence intervals.

For an alpha level of 0.05, effect size of 0.3, [10] and a power of 0.85, we estimated that a sample size of 100 participants would be required.

A kappa statistic (κ) between 0.01 and 0.20 was designated as “slight agreement”; 0.21 and 0.40 as “fair agreement”; 0.41 and 0.60 as “moderate agreement”; 0.61 and 0.80 as “substantial agreement”; and 0.81 and 0.99 as “almost perfect agreement.” A subsequent analysis was done to determine the performance of the working optometrists versus the optometry students.

## Results

Table 1 presents the demographic characteristics of the study participants. The mean age of the participants was 22 years (range: 19–43 years), 87% being women. Forty-five (90%) of those practicing had completed Bachelor’s degree in Optometry, having undergone an average of 4 years (range: 2–7 years) of optometry education. Of those who responded (n = 37) to the question about “years of experience,” about 57% (21/37) of optometrists reported as having less than 2 years of optometry experience. A majority (91%) reported of having seen a fundus photograph before, and 80% reported as not having prior experience in grading DR.

Table 2 presents the agreement between optometry participants versus retina specialist for DR for the 3 referral criteria. Optometry participants correctly identified 91.5% images that required immediate referral (κ = 0.696). Similarly, they correctly identified 62.5% of images as requiring review after 6 months (κ = 0.462) and 51.2% of those requiring review after 1 year (κ = 0.532).

**Table 2:** The Number of Correct and Misclassified Images in Each Referral Type and the Agreement Between Optometrists Versus Retina Specialists.

	Referral Criteria	Optometry Group Referral				Kappa ± SE	Diagnostic capability of the Optometrists		
			No	Yes	Total		Sensitivity(%)	Specificity(%)	AUC
							[95% CI]	[95% CI]	[95% CI]
	Immediate referral	No	1857	522	2379	0.696 ± 0.010	91 [90–92]	78 [76–79]	0.855 [0.843–0.866]
**Retina**		Yes	204	2197	2401				
**specialist**		Sub-total	2061	2719	4780				
	Review after 6 months	No	2996	543	3539	0.462 ± 0.014	62 [59–65]	84 [83–85]	0.727 [0.710–0.744]
(gold		Yes	465	776	1241				
standard)		Sub-total	3461	1319	4780				
	Review after 1 year	No	3483	159	3642	0.532 ± 0.015	51 [48–54]	95 [94–96]	0.824 [0.806–0.842]
		Yes	555	583	1138				
		Grand total	4038	742	4780				

AUC, areas under the receiver-operating characteristic curves; CI, confidence intervals

Table 2 also presents the diagnostic performance among the graders. The sensitivity and specificity of the algorithm among optometrists was 91% and 78% for immediate referral, 62% and 84% for review after 6 months, and 51% and 95% for review after 1 year criteria. The AUC was highest (AUC = 0.855) for immediate referral, second highest (AUC = 0.824) for review after 1 year, and 0.727 for review after 6 months criteria.

Table 3 presents the performance of optometry students versus the working optometrists.

**Table 3:** Performance of Working Optometrists versus Optometry Students.

Referral Criteria	Students			Working optometrists			Chisq
	Sensitivity(%)	Specificity(%)	AUC	Sensitivity(%)	Specificity(%)	AUC	p-values
Immediate referral	93%	80%	0.860	91%	77%	0.842	<0.001
Review after 6 months	65%	85%	0.748	60%	84%	0.722	<0.001
Review after 1 year	54%	97%	0.753	49%	95%	0.720	<0.001

AUC, areas under the receiver-operating characteristic curves

The optometry students performed better for all grades of referral than the working optometrists (AUCs were 0.860, versus 0.842 for immediate referral, 0.748 versus 0.722 for review after 6 months and 0.753 versus 0.720 for review after 1 year, p <0.001 for all criteria).

We also compared the diagnostic performance of the participants who reported as having performed DR grading versus as not having performed prior DR grading. For those who had performed prior DR grading, the AUC was observed to be 0.860 (SE = 0.018) and the AUC for those who had not performed prior DR grading was 0.848 (SE = 0.007) and the differences by chi-square test was not significant, p = 0.534.

To assess the effect of learning curve, we divided the dataset into two sets, consisting of 25 images per set. We analyzed the diagnostic performance in each set; the AUC for the first 25 images was 0.835 (SE = 0.009) and AUC for the second set of 25 images was 0.858 (SE = 0.008). The difference in the diagnostic performance between the first set and second set was not statistically significant, p = 0.06.

## Discussion and Conclusions

We report a novel pictorial method for identification of diabetic retinopathy lesions for appropriate time based referrals, namely—immediate, in 6 months, and in one year with a specific focus for referring sight-threatening DR (Or that which need immediate referral). The algorithm tested among optometrists demonstrated 91% sensitivity for images that needed immediate referral. Even though all participants received a uniform training before the commencement of grading, we were interested in determining whether differences exist in the ability of referral between working and student optometrists. We observed that the diagnostic performance of the optometry students was better than that of the working optometrists for all grades of referral. A likely explanation could be that those in training may be more open to learning new concepts compared with the practicing optometrists who may show some resistance or be biased towards concepts acquired with experience.

S1 Table shows the diabetic retinopathy referral guidelines in various nations.

The main differences in these guidelines are the differential referral of moderate and severe nonproliferative DR; few guidelines adopt less than yearly referral while others prefer an immediate referral. Similarly, the referral criteria for diabetic macular edema also differ across studies because of the differences in the definitions used. S1 Table also shows the expected performance of the algorithm in various guidelines. The performance in our study is similar to that used in Vision 2020 India, Scottish DR, and NHMRC Australia guidelines. It is partly similar to that of Pacific Island Nations, New Zealand DR screening, Diabetic Retinopathy Screening Service for Wales, American Academy of Ophthalmology, and the International Council of Ophthalmology guidelines but different from the DR screening program of Ireland.

Previous studies have compared the grading performance of nonophthalmologist versus ophthalmologist. Farley et al[11] observed that using single-field 45 degree nonmydriatic retinal camera, family physicians failed to refer only 10.2% of the 1040 patients who needed referral compared with the referral advice of the ophthalmologist. A sensitivity of 85% to 97% and specificity of 80% to 96% have been reported for trained graders for detecting STDR and referable DR using nonmydriatic retinal images [12,13]. Andonegui et al[14] examined the performance of primary-care physicians in comparison with that of the ophthalmologist in utilizing 5-field nonmydriatic photographs for detecting DR, in a randomized sample of 200 patients. The physicians received an online clinical preparatory education before grading the images. The agreement between primary-care physicians and the ophthalmologist was between 80% and 95% for the detection of DR. Similarly, our study showed sensitivity of 91% and specificity of 78% to detect immediate referrals. However, for yearly follow-up, the sensitivity was lower (51%) but with good specificity (95%). This can be probably owing to erring toward a more frequent follow-up rather a delayed one in less severe DR.

Like the grading algorithm described in the study, there were previous reports of similar simplified grading tools. Rudnisky et al[15] described a web based computer assisted ETDRS algorithm, and found a good agreement for levels of retinopathy and macular edema with standard slide-film stereoscopic 7-field fundus photography. Likewise, Lecleire-Collet et al[16] described DR classification based on visual comparison between three digital color fundus photos and standard retinal photographs and found the diagnosis of severe levels of DR with high sensitivity and specificity (100% and 58% respectively). Aldington et al[17] in the EURODIAB IDDM complication study reported a new system of 45 degree fundus photo field grading which was used by graders. They showed good repeatability and accuracy of the system. Gangaputra et al[18] in the ACCORD Eye study and FIND study described photographic grading scales which were condensed to correspond to clinical scales. The agreement between clinical and the new scale used by graders was 69% (ACCORD eye) and 74% (FIND study).

Over the last few years, the screening of DR has undergone a paradigm shift to digital retinal photography. As the graders are often nonmedical staff, there is a need to have simple algorithms to refer DR for appropriate management. Recently, Brady et al[19] have described an online tool to train non-experts (Amazon mechanical Turk workforce) to diagnose presence or absence of DR. By minimal training they could rapidly and correctly categorize normal versus abnormal. However, this training could not classify the disease to allow referral. The algorithm described in our study and validated among optometrists can be a simple and accurate method for referral. The algorithm can be tweaked appropriately to regional referral guideline. It may also be appropriate to test the algorithm among other care givers such as physicians, nurses, and photographers.

The optometrists are now increasingly involved in care of diabetic eye disease as often they are the first contact for people with diabetes. Currently their grading is influenced by their training during their optometry education and can be inconsistent. The new algorithm allows them to have uniform diagnostic and referral platform. A similar unified grading platform is currently available in few telemedicine networks like Joslin vision network, where all graders are licensed optometrists and use Joslin Vision Network (JVN) protocol for grading DR. However, use of these algorithms require validated standardized method of certification and training. The algorithm can also serve as a good teaching tool in both undergraduate and postgraduate curriculum for physicians, ophthalmologists, optometrist and other allied courses for people involved in DR care.

We observed no significant difference in the performance of those who reported as had done prior DR grading compared with those who had not. We observed no statistically significant learning effect, although the participants performed slightly better for the second set of images presented compared to first set of images. In addition, we did not conduct a re-grading of the images but that would have been interesting. We are unable to report the effect of re-grading, but we believe that conducting regular training would improve the performance of the participants.

Our study also shows that about 9% of the participants incorrectly classified the images that required immediate referral and this may be of concern. However, the 91% of participants who had correctly advised referral had no prior experience in grading DR images. In addition, only one didactic lecture and a guided grading of just 10 images may not be sufficient. The participants need more education and training. Therefore, regular frequent training may help to further increase the number of correct referrals. Although our algorithm utilizes single-field photography which is advantageous for a quick screening of large masses, it will not replace clinical examination using indirect ophthalmoscopy.

Over the last few years, the screening of DR has undergone a paradigm shift to digital retinal photography. As the graders are often nonmedical staff, there is a need to have simple algorithms to refer DR for appropriate management. The algorithm described in this work and validated among optometrists can be a simple and accurate method for referral. The algorithm can be tweaked appropriately to regional referral guideline. It may also be appropriate to test the algorithm among other care givers such as physicians, nurses, and photographers.

In conclusion, we describe a simple referral algorithm for DR, which has been validated among optometrists and can be used in DR screening services for accurate referral of sight-threatening DR for further management and care.

## Supporting Information

S1 Dataset (XLSX)

S1 Table Diabetic Retinopathy Referral Guidelines in Various Nations. Abbreviations: DR, diabetic retinopathy; NPDR, nonproliferative diabetic retinopathy; DME, diabetic macular edema; CSME, clinically significant macular edema; NHMRC, National Health and Medical Research Council. Scottish: R0, no DR; R1, mild background DR; R2, referable background DR; R3, referable background; R4, proliferative; R5, enucleated; R6, inadequately visualized; M1, observable maculopathy; M2, referable maculopathy; Wales (DRSSW)- R1, mild background DR; R2, preproliferative DR; R3S, stable post treatment; M1, referable maculopathy; R3A,active proliferative DR. New Zealand and Pacific Island Nations: R0, no DR; R1, minimal DR; R2, mild DR; R3, moderate DR; R4, severe DR; R5, proliferative; RT, stable treated; M0, no maculopathy; M1, minimal; M2, mild; M3, mild; M4, moderate; M5, severe; MT, stable treated; UK-R0, none; R1, background; R2, preproliferative; R3, proliferative; R3S, stable treated proliferative; M0, nonreferable maculopathy; M1, maculopathy. Ireland: R0, no DR; R1, background DR; R3, referable background DR; P1, stable treated DR; P2, unstable treated DR; M0, no maculopathy; M1, referable maculopathy.
(DOCX)

## Abbreviations

AUC: area under receiver-operating characteristic curve
DR: diabetic retinopathy
ETDRS: Early Treatment Diabetic Retinopathy Study
IRMA: Intraretinal microvascular abnormality
LCD: Liquid Crystal Display
NVD: Neovascularization at the disc
NVE: Neovascularization elsewhere
STDR: sight-threatening diabetic retinopathy
