# Supplementary material for: Development and Validation of a Diabetic Retinopathy Referral Algorithm Based on Single-Field Fundus Photography
Source: PLoS One. 2016 Sep 23;11(9):e0163108. doi: 10.1371/journal.pone.0163108 (PMC5035083; doi:10.1371/journal.pone.0163108)
Supplement: S1 Table — Abbreviations: DR, diabetic retinopathy; NPDR, nonproliferative diabetic retinopathy; DME, diabetic macular edema; CSME, clinically significant macular edema; NHMRC, National Health and Medical Research Council. Scottish: R0, no DR; R1, mild background DR; R2, referable background DR; R3, referable background; R4, proliferative; R5, enucleated; R6, inadequately visualized; M1, observable maculopathy; M2, referable maculopathy; Wales (DRSSW)- R1, mild background DR; R2, preproliferative DR; R3S, stable post treatment; M1, referable maculopathy; R3A,active proliferative DR. New Zealand and Pacific Island Nations: R0, no DR; R1, minimal DR; R2, mild DR; R3, moderate DR; R4, severe DR; R5, proliferative; RT, stable treated; M0, no maculopathy; M1, minimal; M2, mild; M3, mild; M4, moderate; M5, severe; MT, stable treated; UK-R0, none; R1, background; R2, preproliferative; R3, proliferative; R3S, stable treated proliferative; M0, nonreferable maculopathy; M1, maculopathy. Ireland: R0, no DR; R1, background DR; R3, referable background DR; P1, stable treated DR; P2, unstable treated DR; M0, no maculopathy; M1, referable maculopathy. (DOCX) [file pone.0163108.s002.docx]

**S1 Table. Diabetic Retinopathy Referral Guidelines in Various Nations**

| **Referral Guidelines** | **Yearly Follow-Up** | **Less Than Yearly Follow-Up** | **Immediate Referral** | **Expected Algorithm Performance** |
| --- | --- | --- | --- | --- |
| Vision 2020 India | No DR, mild NPDR, no DME | Moderate NPDR | Severe NPDR, PDR, DME (mild, moderate, and severe) | Similar |
| International Council of Ophthalmology | No DR, mild NPDR | Moderate NPDR, DME (not center involving), severe (within few months) | PDR, DME (center involvement) | Partly similar, but severe is not an immediate referral |
| American Academy of Ophthalmology | No DR, min DR, mild NPDR | Moderate NPDR, severe NPDR, macular edema with no CSME, nonhigh-risk PDR | CSME, high-risk PDR | Partly similar, but severe is not an immediate referral |
| Scottish Diabetic retinopathy grading scheme | R0, R1, R5 | R2, M1 | R3, R4 and R6, M2 | Similar |
| Diabetic Retinopathy Screening Service for Wales (DRSSW) | None, R1, R2, and R3S (after secondary grading) and M0 |  | M1, R3A | Similar for DR, macular edema different |
| New Zealand Diabetic Retinal Screening Guidance | R0, R1, and R2, M0 and MT (biennial), M1 | R3, R4, M2, M3 | R5, RT, M4, M5 | Similar for macular edema, not for DR (as moderate is referable) |
| UK Diabetic Eye Retinopathy Grading Criteria | R0, R1, M0 | R3S (surveillance) | R2, R3, M1 | Similar for DR and macular edema except R3S and M0 |
| NHMRC Australia | No DR (indigenous), mild NPDR, nonindigenous (biennial) | Moderate NPDR | PDR and DME | Similar except no DR in nonindigenous |
| Diabetic Retinopathy Screening Program for Ireland | R0, M0, stable treated DR |  | R1, R2, R3, M1, unstable treated DR | Not similar for both DR and DME |
| Pacific Island Nations | R0, R1, M0, M1, MT | R2, RT, M2 (2 months), M3 (1 month) | R3, R4, R5, M4 | Partly similar for DME and DR |

Abbreviations: DR, diabetic retinopathy; NPDR, nonproliferative diabetic retinopathy; DME, diabetic macular edema; CSME, clinically significant macular edema; PDR, proliferative diabetic retinopathy. NHMRC, National Health and Medical Research Council.

Scottish: R0, no DR; R1, mild background DR; R2, referable background DR; R3, referable background; R4, proliferative; R5, enucleated; R6, inadequately visualized; M1, observable maculopathy; M2, referable maculopathy; Wales (DRSSW)- R1, mild background DR; R2, preproliferative DR; R3S, stable post treatment; M1, referable maculopathy; R3A,active proliferative DR. New Zealand and Pacific Island Nations: R0, no DR; R1, minimal DR; R2, mild DR; R3, moderate DR; R4, severe DR; R5, proliferative; RT, stable treated; M0, no maculopathy; M1, minimal; M2, mild; M3, mild; M4, moderate; M5, severe; MT, stable treated; UK-R0, none; R1, background; R2, preproliferative; R3, proliferative; R3S, stable treated proliferative; M0, nonreferable maculopathy; M1, maculopathy. Ireland: R0, no DR; R1, background DR; R3, referable background DR; P1, stable treated DR; P2, unstable treated DR; M0, no maculopathy; M1, referable maculopathy.
